# An SNP-Based Linkage Map for Zebrafish Reveals Sex Determination Loci

**DOI:** 10.1534/g3.111.000190

**Published:** 2011-06-01

**Authors:** Kevin M. Bradley, Joan P. Breyer, David B. Melville, Karl W. Broman, Ela W. Knapik, Jeffrey R. Smith

**Affiliations:** * Department of Medicine, Vanderbilt University School of Medicine, Nashville, TN 37232-0275; † Department of Cell and Developmental Biology, Vanderbilt University School of Medicine, Nashville, TN 37232-0275; ‡ Department of Biostatistics and Medical Informatics, University of Wisconsin, Madison, WI 53706; § Department of Cancer Biology, Vanderbilt University School of Medicine, Nashville, TN 37232-0275

**Keywords:** *DMRT1*, doublesex, *CYP21A2*, 21-hydroxylase, sex determination, teleost fish, SNP, genetic map, recombination rate, genetic diversity, structure, zebrafish

## Abstract

A surprising diversity of mechanisms controls sex determination of vertebrate organisms, even among closely related species. Both genetic and temperature-dependent systems of sex determination have been described in teleost fish. In the common zebrafish model organism, heteromorphic sex chromosomes are not observed, and the potential role of a genetic component of sex determination remains largely unknown. Here we report a genome-wide linkage study of sex determination in zebrafish using a novel SNP genetic map. We identified loci on zebrafish chromosomes 5 (LOD score 7.9) and 16 (LOD score 9.3) governing sex determination as a complex trait, rather than as an XY or ZW genetic system. Each of these loci contains a prominent candidate gene with a conserved role in sex determination across additional species that suggest potential mechanisms of sex determination in zebrafish. The chromosome 5 locus harbors *dmrt1*, a key gene in sex determination from fruit flies to humans; mutation of the human *DMRT1* ortholog is a cause of complete sex reversal of XY individuals. The chromosome 16 locus harbors *cyp21a2*; mutation of the human *CYP21A2* ortholog is one of the more common causes of pseudohermaphroditism. Mutation detection at each of these candidate genes within the zebrafish cross identified hypomorphic variants on the female-associated allele of each locus. The two loci together accounted for 16% of variance of the trait. Interacting environmental cues are likely to be an additional important component of sex determination in zebrafish.

Over the prior two decades, the zebrafish has become an increasingly important vertebrate model organism. The power of this model organism as a genetic system is notable; with thousands of progeny in a single generation, the large number of recombination events can lead to the identification of loci underlying a trait with high statistical confidence. In this study, we sought to identify genes contributing to sex determination in zebrafish by genome-wide linkage analysis employing a novel SNP genetic map. No prior investigation has elucidated genetic sex determination (GSD) or identified sex-linked markers in the zebrafish. Although the zebrafish does not have heteromorphic sex chromosomes (Amores and Postlethwait 1999; Sola and Gornung 2001), the possibility of homomorphic sex chromosomes such as the X and Y of medaka fish remained (Matsuda *et al.* 2002). Alternatively, sex determination in zebrafish might result from the interaction of a more complex genetic system with environmental factors such as temperature. In some species with temperature-dependent sex determination (TSD), key required genes prove to be the same as those required for GSD (Ferguson-Smith 2007; Marshall Graves 2008; Shoemaker-Daly *et al.* 2010). The mechanisms for their control appear to have evolved distinctly. Because different species of fish have evolved that represent both XY and ZW GSD, as well as TSD mechanisms, fish are particularly suited to the study of sex determination.

## Materials and Methods

### SNP discovery

Methods for SNP discovery have previously been described elsewhere in detail (Bradley *et al.* 2007). In brief, non-unique regions of the zebrafish genome assembly were identified using the BLAST algorithm to create a zebrafish repeat database. Clone sequence and whole genome shotgun sequence produced by the *Danio rerio* Sequencing Group at the Sanger Institute was obtained from online databases. Finished clones were masked using the repeat database, and were then compared to shotgun sequence by BLAST to identify sequence mismatches. Each variant was then scored to highlight those candidate SNPs with only two alleles that were seen in similar proportions, and at regions sampled with greater frequency. Polymorphism of candidate SNPs was evaluated within a sample of zebrafish comprised of: 1) the MGH cross G0 fish, 2) one male C32 and one male SJD fish, and 3) four males and four females from each of the strains AB, IN, TL, TU, and WIK. Among 5321 candidate SNPs successfully converting for assay, a score of ≥4 corresponded empirically to an 86% validation rate; a score of ≥6 corresponded to a 93% validation rate. All candidate SNPs of score ≥4 have been submitted to dbSNP (ss48400959-ss49840083 and ss192416505-ss193075580, discontinuous).

### Fish stocks

Frozen AB (n = 8), TL (n = 8), TU (n = 8), and WIK (n = 8) strain fish were provided by the Zebrafish International Resource Center (University of Oregon, Eugene, OR). Frozen IN (n = 8) population fish were provided by the Vanderbilt Zebrafish Core Facility. For each strain, half of the fish were of each sex. The fish of a given strain were offspring from 25 or more group matings of wild-type fish. Each group mating consisted of two females and three males. Frozen fish of each of the partially inbred strains SJD and C32 were provided by Dr. Stephen Johnson. Genomic DNA was extracted using the PureGene Genomic DNA Purification Kit (Gentra Systems, Minneapolis, MN). DNA was quantified by PicoGreen assay (Molecular Probes/Invitrogen, Carlsbad, CA).

### SNP genotyping

SNP genotyping was conducted using the commercial Illumina GoldenGate assay (Illumina, San Diego, CA). We obtained 99.2% of the genotypes for polymorphic SNPs. Five DNA samples from the MGH cross accounted for 99.4% of the missing data. These were omitted from analysis. For informative SNPs within the MGH cross, 446,994 of 451,360 genotypes (99.0%) were successfully obtained.

### Genetic map construction and linkage analysis

The MGH cross design has been previously described (Knapik *et al.* 1996; Knapik *et al.* 1998; Shimoda *et al.* 1999). Briefly, a female AB strain fish originating from the University of Oregon zebrafish facility and a male India (IN) strain fish originating from a collection of wild fish from the northeast of India were mated to produce a single F1 pair, from which 790 F2 progeny were derived. A subset of 520 were selected for which DNA preparations were of high-quality; the 44 F2 progeny employed for the MGH linkage map were a subset of these. MapManager QTXb20 (Manly *et al.* 2001) was initially used to construct a comprehensive map of the SNP and STR markers, based upon the 44 fish with complete genotype data. This employed the Kosambi map function for the line cross and allowed for segregation distortion, with a *P* = 1.0E-6 threshold. R/qtl (Broman *et al.* 2003) was then used to refine marker order and estimate inter-marker distances, using the full set of 520 fish. This genetic map included both STR (1.2 cM resolution) and SNP (0.1 cM resolution) data. Linkage analysis to identify sex-determining loci was also conducted in the R/qtl environment, using a variant of interval mapping appropriate for a binary trait (Broman 2003). Statistical significance for linkage to sex was established via a permutation test (Churchill and Doerge 1994), with 10,000 permutation replicates. Percent phenotypic variance attributable to a locus was estimated by modeling the binary trait as continuous.

Statistically unlikely events such as double crossovers in adjacent intervals or single crossovers in a small interval in both maternal and paternal meioses were identified using a maximum likelihood approach (Lincoln and Lander 1992). A total of 223 SNP genotypes (0.05%) at internal map loci were flagged as potential errors by calculating the LOD_error_. This rate matches the genotyping accuracy of the Illumina system. The SNP with the greatest number of potential errors had nine genotypes flagged. For STRs at internal map loci, a single genotype (0.001%) was flagged as a potential error. No markers were excluded on the basis of potential genotyping error. We noted seven regions in each of which STR marker order may be inverted with equal probability: pairs of vectors represented by STR markers of chromosomes 2 (Z9234 and Z4586, a 0.8 cM interval), 4 (Z9667 and Z45710, a 1.1 cM interval), 8 (Z14917 and Z28258, a 1.1-cM interval), 12 (Z3690 and Z7328, a 2.3-cM interval), 12 (Z9416 and Z22666, a 1.1-cM interval), 18 (Z9154 and Z25764, a 2.0-cM interval), and 25 (Z13622 and Z6924, a 1.1-cM interval). The merged SNP and STR map, and individual genotype vectors have been provided to the Zebrafish Information Network. Additional detail of the map and markers is available at http://dna.mc.vanderbilt.edu/zbase, and in Table S1.

### Recombination rate estimation

We estimated the recombination rate along the chromosomes (in cM per Mb) using a sliding window of 5 Mb, based upon the merged SNP-STR genetic map and the Zv9 draft assembly. The analysis is restricted to markers with concordant chromosomal assignment and order in the genetic and physical maps.

### Mutation detection

Primers for a series of overlapping amplimers were designed such that the amplimers stair-stepped across Zv8 chromosome 5 from 43,945,234 bp to 44,017,451 bp, and chromosome 16 from 15,088,150 bp to 15,121,079 bp. The amplimer sets were filtered for the select subset encompassing *dmrt1* and *cyp21a2* exons, flanking intronic regions, and upstream sequence including the promoters. A BLAST-based algorithm in primer design specified unique amplification within the Zv8 assembly. One male and one female fish of the cross (each homozygous for the respective alternative alleles) were evaluated for variant detection by resequencing. Primer sequences are available from the authors upon request. The identified variants have been submitted to dbSNP (ss184963416-ss189156666 (*dmrt1*), and ss255538733-ss255540354 (*cyp21a2*), discontinuous).

## Results and Discussion

### SNP genetic map of zebrafish

**Figure 1:**
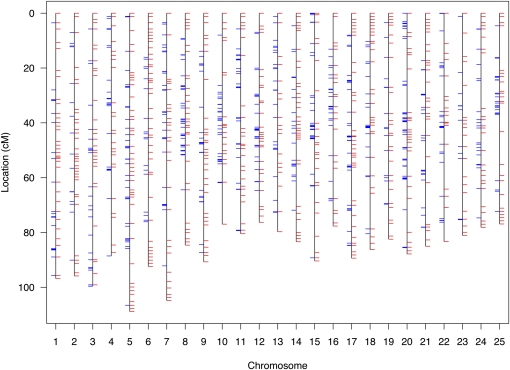
Unified SNP and STR genetic map of the zebrafish. The SNP map is comprised of 870 SNPs at 618 unique loci (blue tick marks) with a mean locus density of 3.4 Kosambi cM and resolution of 0.01 cM. The SNP genotype data were merged with data of 1989 STRs (red tick marks) of the MGH genetic map for creation of a combined map. The merged map is comprised of 1103 unique loci with a mean locus density of 2.0 Kosambi cM. Further detail of map markers and positions is provided in Table S1.

To facilitate genetic mapping of sex determination in zebrafish, we first created an SNP-based framework genetic map. We identified 1,174,978 candidate diallelic SNPs in 10,437 finished clones sequenced by Sanger, roughly one per kilobase of clone sequence. This resource roughly doubled the number of SNPs that we had previously identified (Bradley *et al.* 2007). We selected six candidate variants distributed across each of 875 clones to position the clones within a genetic map. We genotyped the candidate SNPs of the clones in an F2 intercross of 1040 meioses (the original 1996 MGH cross) (Knapik *et al.* 1996; Knapik *et al.* 1998; Shimoda *et al.* 1999). Of SNPs successfully assayed, 82% (3966) validated as polymorphic in the cross or in common laboratory strains (AB, IN, TU, TL, WIK, C32, SJD). The specific grandparental chromosomes of the cross were distinguished by 22% of the SNPs. These anchored a subset of 540 of the 875 clones on the genetic map, often redundantly with multiple informative SNPs per clone, totaling 618 unique loci (Figure 1 and supporting information, Table S1). The 1040 meioses of the cross provided a resolution of 0.1 cM. The mean density of the framework SNP map was 3.4 cM and the largest gap was 26.2 cM. An additional 2005 SNPs (polymorphic in the strains, but not informative in the cross) were located within the clones positioned on the framework genetic map. Thus the general resource for positional cloning in zebrafish is comprised of 2875 positioned SNPs (Table S2). An optimally informative zebrafish SNP genetic mapping panel and evaluation of the genetic ancestry of common laboratory strains are described in Figure S1, Figure S2, and Table S3.

The 44 zebrafish employed for creation of the MGH microsatellite (simple tandem repeat, STR) genetic map were a subset of the 520 employed to create the framework SNP genetic map (Knapik *et al.* 1998; Shimoda *et al.* 1999). This allowed us to subsequently distribute markers of the original MGH map into the higher-resolution SNP map. A total of 241 STRs were positioned at loci that were uniquely identified, and a total of 1748 STRs were positioned at loci that were each redundantly identified (*e.g.*, two STRs with the exact same series of genotypes across the 44 F2 progeny identify the same locus). All STR loci were supported by LOD scores ≥7. This strategy conservatively omitted markers with lesser statistical support in map construction. We observed no discordance of STR marker chromosomal assignment or order between this merged map and that of the original MGH map. The merged SNP-STR map has a mean genetic locus density of 2.0 cM, and largest gap of 16.0 cM (Figure 1 and Table S1).

### Zebrafish recombination rate estimation

**Figure 2:**
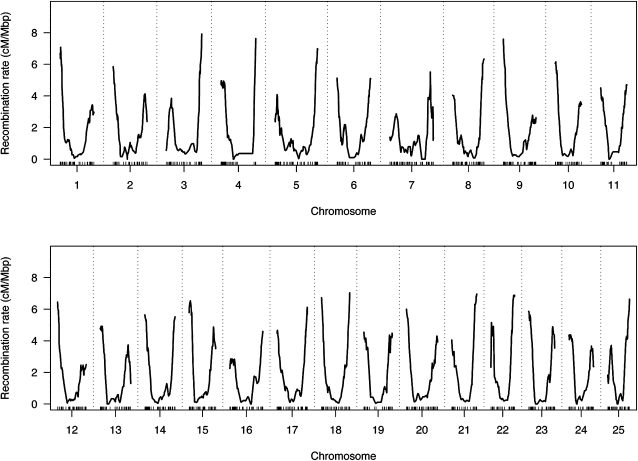
Recombination rate across the zebrafish genome.

We evaluated recombination rate across the zebrafish genome based upon this map, presented in Figure 2. We observed a mean recombination rate of 1.60 cM/Mb for the zebrafish genome. By comparison, the mean sex-averaged recombination rate is 1.13 cM/Mb for human, and 0.63 cM/Mb for the mouse (Kong *et al.* 2002; Shifman *et al.* 2006). A high rate of recombination at all distal chromosomal arms is especially pronounced in zebrafish. Telomeric recombination rates reach roughly 2.5 cM/Mb in mouse, while rates often exceed 4 cM/Mb in zebrafish. Distal telomeric recombination suppression is also visible for many chromosomal arms.

### Concordance of zebrafish genetic and physical maps

A comparison of the genetic map to the Zv9 physical map revealed that few markers were assigned to different chromosomes (1.4% of SNPs and STRs, 0.6% of SNPs, 1.0% of clones). We calculated LOD scores comparing the location of each of these markers in the assigned genetic chromosome to any location on the assigned physical chromosome. Genetic map position support LODs ranged from 12 to 228, while the physical map position support LODs ranged from 0 to 0.1.

Other markers were positioned with a different relative order within a given chromosome of the genetic and Zv9 physical maps (7.2% of SNPs and STRs, 3.6% of SNPs, 5.8% of clones). We calculated LOD scores for each chromosome, comparing the marker order in the physical and genetic maps. These LOD scores ranged from −0.03 to −319 (supporting genetic map order). Seventeen percent of the clones genetically positioned by SNPs were oriented by virtue of informative intra-clone recombination events in the cross; 13% of these had relative inverse orientations within the genetic and physical maps. Table S1 provides both genetic and physical map positions of each marker. Alternative explanations for the subset of discordantly positioned markers include error in either map, or large-scale structural polymorphism among different zebrafish populations. Structural polymorphism within the repetitive genome could confound physical assembly based upon clones of many individuals of a population, and could also fail to be evident within a genetic map based upon a single cross.

### Genome-wide linkage analysis of zebrafish sex

**Figure 3:**
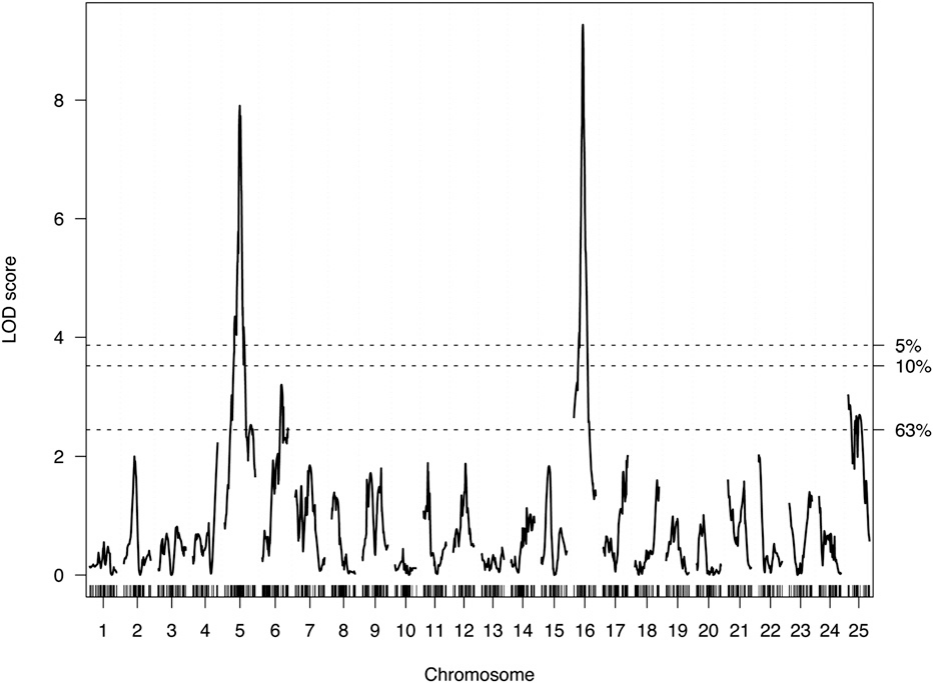
Multipoint interval mapping of sex determination in zebrafish. LOD score results are plotted as a function of marker location in cM, with chromosomal number designated at the bottom of the plot. Dashed horizontal lines indicate significance thresholds (α) determined by permutation testing.

**Figure 4:**
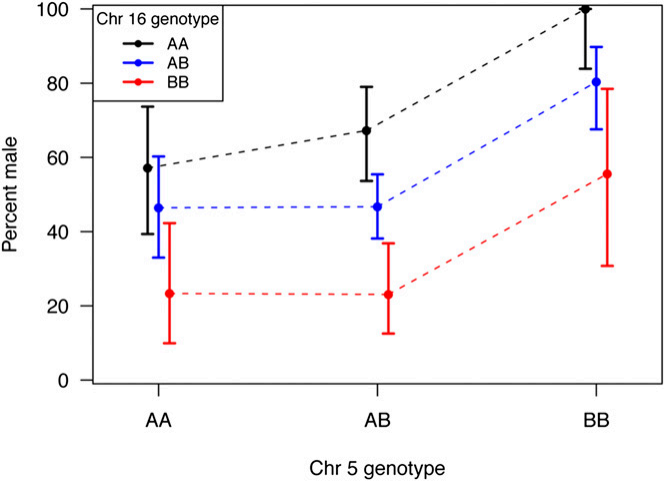
Plot of the proportion of male progeny of the cross as a function of the alleles at the two major sex-determining loci in zebrafish. The B allele of chromosome 5 (originating from the IN strain male grandparent) corresponds to the male-associated allele of *dmrt1*, here marked by ss48808156 (T variant of the A/T SNP) in the cross. The A allele of chromosome 16 (originating from the AB strain female grandparent) corresponds to the male-associated allele of *cyp21a2*, here marked by ss48940970 (T variant of the T/C SNP) in the cross. The 95% confidence intervals of the proportion of males among zebrafish with a given two-locus genotype are illustrated for each data point.

The mapping cross provided substantial power to identify loci controlling sex determination in zebrafish. Of the 520 F2 sibling progeny, 219 were female and 248 were male (53 were of unknown sex). LOD scores for linkage to sex-determination identified two loci that exceeded genome-wide significance for linkage to sex (Figure 3), with nearly identical results for SNP or merged SNP-STR data. One locus resided on chromosome 5 at 53.8 cM with an LOD of 7.9 [near ss48808156 at 53.3 cM, 1.5 LOD drop interval ss48660708 (49.1 cM) to ss48502349 (61.1 cM)]. The other resided on chromosome 16 at 32.0 cM with a LOD of 9.3 [near ss48940970 at 30.4 cM, 1.5 LOD drop interval ss192546947 (28.9 cM) to ss48768171 (35.0 cM)]. More minor peaks are visible on chromosomes 6 and 25 (near ss192942334 and ss48734451, respectively), but neither reached statistical significance. Although these LOD scores were notably large, the LOD score anticipated in this cross if there were a single fully penetrant sex determination gene would have been ∼140. This contrast to the observed LODs highlights the complexity of sex determination in zebrafish. The loci on chromosomes 5 and 16 each explain a fraction of trait variance (7% and 9%, respectively). There was no evidence for an epistatic interaction between the two loci; together they are estimated to account for 16% of the trait variance. Alleles of the chromosome 5 locus appear to act in a recessive male (or alternatively, dominant female) fashion. Each allele of the chromosome 16 locus appears to act additively (one allele male, the other female). Figure 4 illustrates progeny sex relative to genotype combinations at these loci in the cross. All progeny (100%) that inherited fully male allele doses of these two loci were male; 77% of progeny that inherited fully female allele doses of these two loci were female (see also Table S4).

### Mutation detection of chromosome 5 locus candidate *dmrt1*

The closest flanking markers of the chromosome 5 sex-determining locus (ss48697105 and ss48960192) define a 1.7-cM interval corresponding to 2.2 Mb (Zv9 chromosome 5: 44,453,011 to 46,626,084 bp). Although many genes reside within this interval, one in particular was a candidate of immediate relevance: *dmrt1*. *dmrt1* is the zebrafish ortholog of the *doublesex* gene, a bifunctional gene regulating both male and female sexual differentiation of the fruit fly. *dmrt1* orthologs are the sex-determining genes of the Y chromosome of medaka and of the Z chromosome of birds (Matsuda *et al.* 2002; Smith *et al.* 2009). In humans, DMRT1 operates downstream of SRY in sex determination; haploinsufficiency of *DMRT1* at 9p24.3 in humans results in sex-reversal of XY individuals (analogous to the recessive male model that we observed in zebrafish) (Muroya *et al.* 2000). Further, *dmrt1* expression is governed by environmental temperature in some species with TSD (Shoemaker *et al.* 2007).

In order to identify a *dmrt1* allele that might guide sex determination in zebrafish, we resequenced the male- and female-associated alleles of *dmrt1* within the cross. They were distinguished by a total of 162 SNPs, 28 indels, and two simple tandem repeats. We recognized two of these as functional candidates: the nonsynonymous D259E [ss184964113 at Zv9 chromosome 5: 46,608,201 bp, not in a highly conserved region of the protein (NP_991191)], and the central nucleotide of the *cis*-regulatory motif of the 3′ UTR (ss184964140 at Zv9 chromosome 5: 46,608,515 bp). The evolutionarily conserved 3′ UTR protein-binding motif has been shown to be responsible for *dmrt1* transcript stability and translational efficiency, restricted to male gonad and germ cell development (Guo *et al.* 2005; Herpin *et al.* 2009). Mutagenesis data that defined the motif predicts that the female-associated allele, 5′-CUGCUACAGAU-3′, would yield lower *dmrt1* expression relative to the male allele 5′-CUGCUGCAGAU-3′ in the developing gonadal primordium. Given the striking conservation of the role of *dmrt1* in sex determination across evolutionary time, and linkage and mutation detection evidence, *dmrt1* is a particularly good candidate of the chromosome 5 locus of the zebrafish cross.

### Mutation detection of chromosome 16 locus candidate *cyp21a2*

The closest flanking markers of the chromosome 16 sex-determining locus, ss48940970 and ss192790632, define a 3.5 cM interval corresponding to 4 Mb (Zv9 chromosome 16: 12,952,287 to 16,952,809 bp). Although many genes reside within the chromosome 16 locus, we recognized one in particular that is salient to the trait. The *cyp21a2* (LOC793249) gene is 1.7 Mb from ss192790632. Mutations of the human ortholog of *cyp21a2* cause an inborn error of sex development (pseudohermaphroditism). *cyp21a2* encodes 21-hydroxylase, biosynthesizing corticosteroids. Cortisol exposure, as well as high temperature, can induce female-to-male sex inversion of genetically female (XX) medaka (Hayashi *et al.* 2010) and Japanese flounder (Yamaguchi *et al.* 2010). Furthermore, pharmacologic inhibition of corticosteroid biosynthesis prevents temperature-mediated inversion of genetically female medaka (Hayashi *et al.* 2010) and Japanese flounder (Yamaguchi *et al.* 2010) into males. In the protogynous grouper, 21-hydroxylase is activated in the transition of ovarian to testicular tissue during exogenous testosterone-mediated female-to-male sex inversion (Lee *et al.* 2002). Collective evidence suggested that *cyp21a2* activity might guide sex determination of the developing zebrafish embryo.

We resequenced the male- and female-associated alleles of *cyp21a2* within the cross seeking variants that might influence 21-hydroxylase activity. The male- and female-associated *cyp21a2* alleles were distinguished by a total of 176 SNPs and 24 indels. Potential copy number variation of the *cyp21a2* gene (as can occur in humans) did not appear to confound variant discovery. Fish homozygous for the male or for the female alleles of the cross were homozygous at (and distinguished by) each of the discovered genetic variants. The female and male alleles differed at four amino acid positions conserved among orthologs of additional teleost fish (Figure S3). The male allele corresponded to the conserved residue at each of the four positions (male/XP_001919231 residue position/female: G141R (ss255539489 and ss255539495), V375M (ss255540264), Y380H (ss255540270), and V527A (ss255540330, Zv9 chromosome 16: 18,639,369 bp), suggesting that the female allele may have reduced function. V527 was additionally invariant across the mouse and human orthologs. These results are consistent with a potential role of relative 21-hydroxylase activity in ovarian or testicular fate specification in the zebrafish cross.

## Conclusions

In conclusion, we developed an accurate SNP genetic map of the zebrafish and applied it to identify a genetic component of sex determination. Our linkage results demonstrate that sex determination is a complex trait in zebrafish, not employing sex chromosomes. A number of candidate genes have previously been investigated with a role in sex differentiation in zebrafish, including: *dnd* (Zv9 chr 14), *fancl* (chr 13), *cyp19a1a* and *b* (chr 18 and 25), *ff1a - d* (chr 22, 8, 3, and 21), *foxL2* (chr 15), *sox9a* and *b* (chr 12 and 3), *wt1a* and *b* (chr 25 and 18), and *amh* (chr 22) (Jorgensen *et al.* 2008; Kuo *et al.* 2005; Onichtchouk *et al.* 2003; Rodriguez-Mari *et al.* 2010; Rodriguez-Mari *et al.* 2005; Schulz *et al.* 2007; Siegfried and Nusslein-Volhard 2008; Trant *et al.* 2001; Uchida *et al.* 2004; Von Hofsten and Olsson 2005; Weidinger *et al.* 2003). Alleles at these loci did not statistically significantly contribute to sex determination within the cross. Instead, we identified two other loci on chromosomes 5 and 16 that statistically significantly contributed to sex determination.

Zebrafish *dmrt1* resides within the chromosome 5 locus; orthologs of this gene play key roles in sex determination of *D. melanogaster*, *C. elegans*, medaka fish, birds, and humans. A variant within the *dmrt1* 3′UTR regulatory element distinguished the male- and female-associated alleles of the zebrafish cross. Zebrafish *cyp21a2* resides within the chromosome 16 locus. Current evidence among several fish species supports a key role for steroid hormones, both sex steroids and corticosteroids, in sex determination. Reduced 21-hydroxylase activity results in reduced corticosteroid biosynthesis, potentially resulting in a shunt of precursors toward sex steroid biosynthesis. Mendelian mutations of the human *CYP21A2* ortholog are a cause of virilization of males and of females (pseudohermaphroditism). Mutations of human *CYP21A2* result in androgen excess, the most common cause of ambiguous external genitalia in the newborn. However, the predicted hypomorphic zebrafish allele (concurrent change of four evolutionarily-conserved amino acids) is that of the female, rather than the male. Prior experiments demonstrating a role for corticosteroids in male development of both medaka and flounder are consistent with a male-associated zebrafish allele of preserved 21-hydroxylase activity, and female-associated zebrafish allele of reduced activity. Corticosteroid-mediated transcriptional suppression of genes that are active in female fish development, such as *fshr* (Hayashi *et al.* 2010), might underlie the apparent paradox between the human and the fish systems of sex determination. Alternatively, testosterone excess (resulting from reduced CYP21A2 activity) as a precursor may be converted to estradiol by aromatase, rather than to active dihydrotestosterone by steroid 5-alpha reductase. It is conceivable that the former pathway may predominate in zebrafish. Note that 17-alpha methyltestosterone treatment of embryonic zebrafish skews to male development (Westerfield 1994), but the synthetic androgen is also an aromatase inhibitor (Fenske and Segner 2004).

Because the two loci each contribute a relatively small proportion of the variability of this trait, experimental demonstration of causality of the identified alleles may be difficult due to expected incomplete effects on biological sex determination. Based upon current knowledge, complete abrogation of function of each candidate gene may have marked effects on sex determination. However, that would not conclusively establish the identified alleles of either gene as the source of the linkage signal within the cross. Another experimental limitation is posed by the observed complex nature of this trait. Traditional interval mapping is suitable for positional cloning of Mendelian mutations, but would not be fruitful here because a given fish with a recombination event that might narrow a critical interval may, due to incomplete penetrance, be of either sex. Lack of one-to-one correlation between the trait and either locus would confound such an effort. Given current evidence, both *dmrt1* and *cyp21a2* are very good candidates as genes potentially underlying the linkage signal of the cross, and further work is warranted to evaluate biological causality. Within the AB and IN strains, as well as other zebrafish populations, additional alleles of these and other genes may be found that collectively account for a greater extent of the variability of this complex trait. The SNP mapping panel derived from our work may be useful in further investigation of additional crosses. Additional complex genetic factors and environmental cues are likely to interact in zebrafish sex determination, and remain to be elucidated.

## Supplementary Material

Supporting Information
