# Supplementary material for: An SNP-Based Linkage Map for Zebrafish Reveals Sex Determination Loci
Source: G3 (Bethesda). 2011 Jun 1;1(1):3–9. doi: 10.1534/g3.111.000190 (PMC3178105; doi:10.1534/g3.111.000190)
Supplement: Supporting Information [file supp_1_1_3__index.html]

Supporting Information 

# An SNP-Based Linkage Map for Zebrafish Reveals Sex Determination Loci

## Supporting Information for Bradley *et al.*, 2011

**Files in this Data Supplement:**

- Supporting Information - Figures S1-S3, File S1, and Tables S1-S4 (PDF, 3 MB)
- Figure S1 - Zebrafish SNP mapping panel (PDF, 3.5 MB)
- Figure S2 - Estimated population structure of common laboratory zebrafish strains (PDF, 116 KB)
- Figure S3 - Constraint-based multiple-alignment of 21-hydroxylase (cyp21a2) (PDF, 1.9 MB)
- File S1 - Supporting Text (PDF, 88 KB)
- Table S1 - SNP and STR positions in the genetic map, and in the Zv9 physical assembly (Microsoft Excel, .xls, 328 KB)
- Table S2 - SNPs of clones positioned in the genetic map, and observed alleles by strain (Microsoft Excel, .xls, 1 MB)
- Table S3 - A mean 4 cM density SNP mapping panel for zebrafish (Microsoft Excel, .xls, 248 KB)
- Table S4 - Count and proportion of male and female F2 progeny in each genotypic category of the chromosome 5 and 16 sex-determination loci (see Figure 4) (Microsoft Excel, .xls, 32 KB)
