# Supplementary material for: An SNP-Based Linkage Map for Zebrafish Reveals Sex Determination Loci
Source: G3 (Bethesda). 2011 Jun 1;1(1):3–9. doi: 10.1534/g3.111.000190 (PMC3178105; doi:10.1534/g3.111.000190)
Supplement: Supporting Information [file supp_1.1.3_FigureS1.pdf]

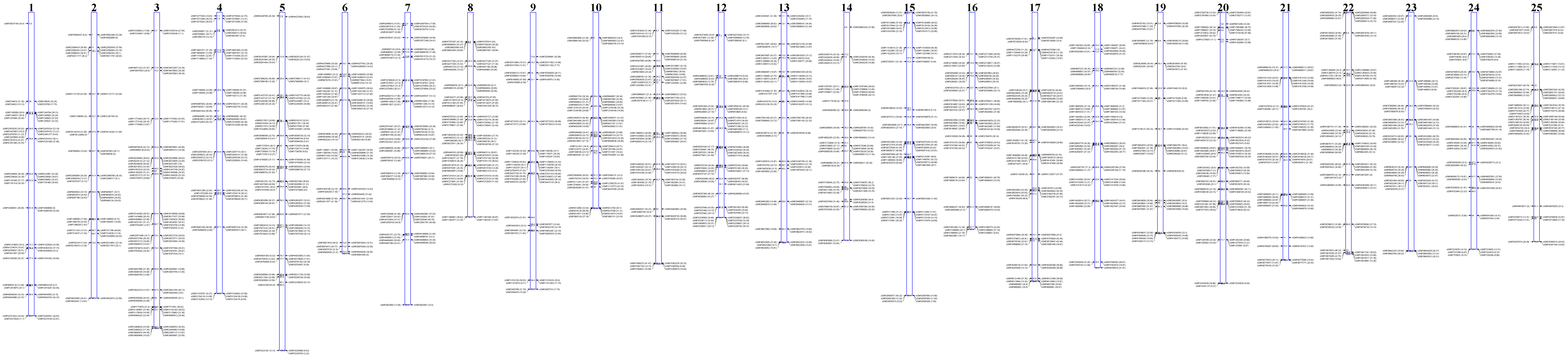

1
2
3
4
5
6
7
8
9
10
11
12
13
14
15
16
17
18
19
20
21
22
23
24
25
