# Supplementary material for: An SNP-Based Linkage Map for Zebrafish Reveals Sex Determination Loci
Source: G3 (Bethesda). 2011 Jun 1;1(1):3–9. doi: 10.1534/g3.111.000190 (PMC3178105; doi:10.1534/g3.111.000190)
Supplement: Supporting Information [file supp_1.1.3_FigureS2.pdf]

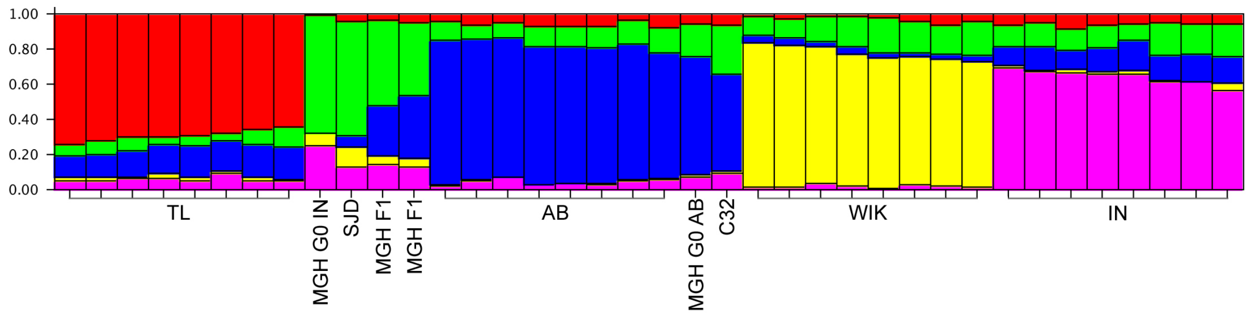

Figure S2 Estimated population structure of common laboratory zebrafish strains. Individual fish were genotyped for 2,875 genetically positioned SNPs for analysis by STRUCTURE under a linkage model. Each individual fish is represented by a vertical bar, which is partitioned into *K* colored segments that represent the estimated membership fraction from *K* ancestral populations. The highest probability model is presented, *K* = 5. Strain membership is designated at the bottom. The G0 grandparents (one AB, one IN) and F1 parents of the MGH cross are included for comparison. Prior information of strain membership was not employed in the analysis.
