# Supplementary material for: An SNP-Based Linkage Map for Zebrafish Reveals Sex Determination Loci
Source: G3 (Bethesda). 2011 Jun 1;1(1):3–9. doi: 10.1534/g3.111.000190 (PMC3178105; doi:10.1534/g3.111.000190)
Supplement: Supporting Information [file supp_1.1.3_FigureS3.pdf]

```
                                             L          K R    K                   P
Danio_rerio             1  1    ----MCFSVVSVVPLLFILWMLVVRFWRQSHRRTDGVAKENNKPEAVFPKLLHSLYKLFFSTVSPTISGPRSLPLLGNML  76
Tetraodon_nigroviridis  2  1    MSVEVLVAGVGAGFLLVLLLMVLKDMCVKKNHNL-----SFSAPPAVKRSLLQSLCGLLHRPSSPSIPGPPCRFLIGNMT  75
Fugu_rubripes           3  1    MVGKHLID--------------LDRMCSFLFCS------SFSAPPAVKRNLPLSLWQLPLRPSSPPIPGPPCRFLIGNMT  60
Gasterosteus_aculeatus  4  1    MVIEISFITAGAAFLVVLL-MIFIGIFCQKDQSV-----QGDHRGKAASGLLHHLCRFLTRSSSPGLPGPPSLFLIGNMM  74
Oryzias_latipes         5  1    ------------------------------------------------------------------------------MM  2
Anguilla_japonica       6  1    MPVCMTFVGAGTAVVLCVLWLVFVYYPRQTDQK---LCSQSN---VVGSTLLYPLNRLLPHTLSPSLPGPPSLPLLGNML  74
Homo_sapiens            7  1    MLL------LGLLLLLPLL---------------------------AGARLLWNWWKL------RSLHLPPLAPGFLHLL  41
Mus_musculus            8  1    MLL------PGLLLLLLLL---------------------------AGTRWLWGQWKL------RKLHLPPLAPGFLHFL  41

                                                                                       H        R
                        1  77   DLAQDHLPIHLTALAKCYGNIYRLNCGSTTMVVLNNSEIIREALVKKWSDFAGRPYSYTGDIVSGGGRTISLGDFSEEWK  156
                        2  76   ELMQDHLPIHLTDLAKRYGNIYRLKCGNTTMVVLSSGDVIREALVKKWSDFAGRSVSYTADIVSGGGRTISLGDYTEEWK  155
                        3  61   ELMHDHLPIHLTNLAKRYGNIYRLKCGNTTMIVLNSSDIIREALVKKWSDFAGRAVSYTADIVSGGGRNISLGDYTEEWK  140
                        4  75   ELTHEHLPIHLTNLARRYGNIYRLKCGNTTMVVLNSGEIIREALVKKWSDFAGRPVSYTGDIVSGGGHTISLGDYNEEWR  154
                        5  3    ELMHDHLPIHLTSLAQRYGSIYRLKCGNTTMVVLNSGELIRETLVRKWSDFAGRPISYTGDIVSGGGRNISLGDYTEEWR  82
                        6  75   ELTRDHLPNHLTSLARHYGNIYRLYCGNTTIVVLSSAEMIREALVKKWSDFAGRPHSYTGDIVSGGGRSISLGDYSEAWR  154
                        7  42   Q---PDLPIYLLGLTQKFGPIYRLHLGLQDVVVLNSKRTIEEAMVKKWADFAGRPEPLTYKLVSRNYPDLSLGDYSLLWK  118
                        8  42   Q---PNLPIYLLGLTQKLGPIYRIRLGMQDVVVLNSNRTIEEALIQKWVDFAGRPHMLNGKMDL----DLSLGDYSLMWK  114

                        1  157  AHRRVTHSALQRCTTDSLHSVIEKQAQHLCQVLRDYSGKAVDLSEDFTVASSNVITTLTFSKAYDKSSAELQKLQECLNE  236
                        2  156  AHRRLVHSALQRCCKQSLHDVIERQALQLRKVLINYQGGAVDLSEDFTVAASNVITTLVFGKEYDKSCSELQQLHSCLNE  235
                        3  141  ALRRLVHGALQRCCKHSLHNVIERQALQLRKVLVDYRGGAVDLSEDFTVAASNVIITLVFGKEYDKSSSELQQLHRCLNE  220
                        4  155  AHRRLVHSALQRCCQQSLHDVIERQALHLREVLMDYRGSPVDLAEDFTVAASNVITTLAFGKEYGKSSSELQKLHSCLNE  234
                        5  83   AHRRLVHGALQRCCQQSLHSVIERQALHLRKVLMGYQGSPVDLSEDFTVAASNVITTLAFGKEYDKSSSELQQLHSCLNE  162
                        6  155  THRRLAHSALQRCSAQCLHAVIQKQALGLRQVLMDYNETPVDLSEDFTVAASNVITTLVFGKEYDKSSPELQRLHGCLNE  234
                        7  119  AHKKLTRSALLLGIRDSMEPVVEQLTQEFCERMRAQPGTPVAIEEEFSLLTCSIICYLTFGDKI-KDDNLMPAYYKCIQE  197
                        8  115  AHKKLSRSALMLGMRDSMEPLIEQLTQEFCERMRAQAGTPVAIHKEFSFLTCSIISCLTFGDK---DSTLVQTLHDCVQD  191

                                                                                       -
                        1  237  IVSLWGSPWISALDSFPLLRKFPNPPFSRLMKEVARRDELIGKHIEEFKVRQIVQIISTESCQKFSKGGTLTSSLLKCLE  316
                        2  236  IVALWGSAWISALDTFPLLRKFPNPVFSRLLREVTRRDEIIRKHLNQYKVQS----------QDNKSTDVITGSLLEGVS  305
                        3  221  IVALWGSTWISALDTFPLLRKFPNPVFSRLLREVSRRDEIIRKHLNQFKVKS----------EGHRRTDVITGSLLEGVD  290
                        4  235  IVALWGSSWISALDSFPLLRKLPNPVFARLLKEVAKRDEIIREHLNNYK--S----------QNNKTEDAITGSLLEGLE  302
                        5  163  IVSLWGSSWISALDSFPLLRKLPNPVFARLLKEVARRDEIIKQHLDNFK--L----------QGHQSEDSITGSLLQALD  230
                        6  235  IVSLWGSPWISALDSFPLLRKLPNPPFSRLLKEVARRDDIIRSHIQEYK----------ELCQDEGTGGTITASLLQSLD  304
                        7  198  VLKTWSHWSIQIVDVIPFLRFFPNPGLRRLKQAIEKRDHIVEMQLRQHKESLV----------AGQWRDMMDYMLQGVAQ  267
                        8  192  LLQAWNHWSIQILTIIPLLRFLPNPGLQKLKQIQESRDHIVKQQLKRHKDSLV----------AGQWKDMIDYMLQGVEK  261

                                                                                          M        H
                        1  317  PQQGAANHTTLTDTHVHMTTVDLLIGGTETIAALLNWTVAFLLHRPEVQDKVYEELCCVLDVR----YPQYSDRHKLPYL  392
                        2  306  KHR-KDHDVVLTDVHVHMATVDLLIGGTETTAAWLNWTVAFLLHRPEIQTKVYEELCTVLEGR----YPKYSDRHRLPVL  380
                        3  291  RHQ-TEPGVVLTDMHVHMATVDLLIGGSETTAAWLNWTVAFLLHRPEFQTKVYEELCTVLEGR----YPKYSDRQRLPIL  365
                        4  303  RNNNTDHKVRLTDIHVHMATVDLLIGGTETTAAWLNWTVAFLLHRPEVQAKVYEELCTVLEGR----YPKYSDRRRLPVL  378
                        5  231  KHQNTKEGQLLTDVHVHMATVDLLIGGTETTAAWLNWTVAFLLHRPEIQDKVYEELCTVPEGR----YPKYSDRQRLPVL  306
                        6  305  QPLGEKERVALTETHVHMATVDLLIGGTETTAAWLGWTVAFLLHRPEVQCRVYSELCTVLDTR----YPQYSDRLKLPCL  380
                        7  268  PXM-EEGSGQLLEGHVHMAAVDLLIGGTETTANTLSWAVVFLLHHPEIQQRLQEELDHELGPGASSSRVPYKDRARLPLL  346
                        8  262  QRD-GKDEERLHEGHVHMSVVDLFIGGTETTATTLSWAVAFLLHHPEIQKRLQEELDLKLGPG---SQLLYRNRMQLPLL  337

                        1  393  CALISEMLRLRPVAPLAVPHRAIRNSSIAGHFIPKNTIIIPNLYGAHHDPEVWDDPYSFKP--ERFLEGGGGSLRSLIPF  470
                        2  381  CSLVHEVLRLRPVAPLAVPHKAVRDSSIAGYFIPKNTIIIPNLFGAHHDPAVWPDPYSFKPVQERFLEGGGGSPRALVPF  460
                        3  366  CSLIHEVLRLRPVAPLAVPHKAIRDSSIAGYFIPRNTIIIPNLFGAHHDPEVWSDPYSFKP--ERFLEGGGGSTRALIPF  443
                        4  379  CSLINEVLRLRPVAPLAVPHRAVRDSSIAGYFIPKNTVIIPNLFGAHHDPAVWSDPYSFRP--ERFLEGGESSTRALMPF  456
                        5  307  CALVNEVLRLRPVAPLAVPHRAIRNSSIAGFFIPKNSVIIPNLFGAHHDPAVWSEPYSFKP--ERFLEGGGGSTRALVPF  384
                        6  381  SALINEVLRLRPVAPLAVPHRAIRDSSIAGYRIPKDTVIIPNLYGAHHDPAMWIDPYAFKP--ERFLDGGRASTRALLPF  458
                        7  347  NATIAEVLRLRPVVPLALPHRTTRPSSISGYDIPEGTVIIPNLQGAHLDETVWERPHEFWP--DRFLEP--GKNSRALAF  422
                        8  338  MATIAEVLRLRPVVPLALPHRATRASSISGYDIPKDMVIIPNIQGANLDEMVWELPSKFWP--DRFLEP--GKNPRTPSF  413

                                                                                          A
                        1  471  GGGARLCLGEAVAKMEMFLFTAYLLREFKFLPASKEEPLPELRGV--ASVVLKVKPYTVIAHPR--EQ-------  534
                        2  461  GGGARLCLGETVAKMELFLFTAYLLRDFCFCLPDVEAALPDLRGV--ASVVLKVKAFTVVARPRIRPLS------  527
                        3  444  GGGARLCLGETVAKMELFLFTAYLLRDFCFVLPDSEAPLPDLRGV--ASVVLKIKSFTVIARPR------------  505
                        4  457  GGGARLCLGESVAKMELFLFTAYLLRDFHFIPPENEASLPDLRGV--ASVVLKVKSYTVTARPR--PLT------  521
                        5  385  GGGARLCLGESVAKMELFLFTAYLLRDFQFILPESNVFPPDLRGI--ATVVLKIKPYKVIARPR--PAIGP----  451
                        6  459  GGGARLCLGESIAKMELFLFTAYLLRDFQFLPSGNKETLPDLTGV--ASVVLKVKPFQVIVRPR--PLT------  523
                        7  423  GCGARVCLGEPLARLELFVVLTRLLQAFT-LLPSGD-ALPSLQPLPHCSVILKMQPFQVRLQPRGMGAHSPGQSQ  495
                        8  414  GCGARVCLGEPLARLELFVVLARLLQAFT-LLPPPDGTLPSLQPQPYAGINLPIPPFQVRLQPRNLAPQDQGERP  487
```

Figure S3 Constraint-based multiple-alignment of 21-hydroxylase (cyp21a2). The cyp21a2 orthologs of *Danio rerio* (zebrafish), *Tetraodon nigroviridis* (green spotted pufferfish), *Fugu rubripes* (Japanese pufferfish), *Gasterosteus aculeatus* (stickleback),

Oryzias latipes (medaka), *Anguilla japonica* (Japanese eel), *Homo sapiens*, and *Mus musculus* are aligned for comparison. The male allele of *Danio rerio* identified within the cross is aligned at the top, with concurrent amino acid changes of the female allele designated above. Highlighted positions are allele changes at residues conserved among fish (in blue), and additionally among humans and mice (in yellow).
