# Supplementary material for: An SNP-Based Linkage Map for Zebrafish Reveals Sex Determination Loci
Source: G3 (Bethesda). 2011 Jun 1;1(1):3–9. doi: 10.1534/g3.111.000190 (PMC3178105; doi:10.1534/g3.111.000190)
Supplement: Supporting Information [file supp_1.1.3_FileS1.pdf]

File S1

Supporting Text

Zebrafish SNP Mapping Panel

Each of the 2,854 positioned SNPs was evaluated to determine the proportion of potential crosses of pairs of fish in which the SNP would have been informative, among the genotyped fish of the AB, IN, TL, TU, WIK, C32, and SJD strains. These SNPs are provided in Table S2. Each simulated cross employed a pair of fish of different strains. To identify an optimal mapping panel of 1,536 SNPs, each positioned SNP was ranked according to the proportion of crosses in which it was informative. A first selection sweep was then made, in decreasing rank order, selecting each SNP that was no closer than 3 cM to a previously selected SNP. Subsequent iterative selection sweeps were then made (again in decreasing rank order), each sweep allowing single additional SNPs to be positioned within 3 cM of those SNPs added in prior sweeps (but at least 60 bp apart, to accommodate assay). Beyond the third iterative selection, to preclude addition of uninformative SNPs to a region already populated with ≥ 3 SNPs, only SNPs informative in at least 5% of the crosses were considered. However, for region populated with ≤ 2 SNPs, even a SNP uninformative in the simulated crosses but informative in the MGH cross (a validated SNP) was considered.

The resulting generic SNP mapping panel has a mean locus density of 4.0 cM (largest gap 26.2 cM), presented in Figure S1 and Table S3. These SNPs are well distributed and most informative for crosses of pairs of the common laboratory strains, based upon the fish sampled in our study. Within the mapping panel, each locus is typically redundantly represented by several SNPs to improve the probability that at least one of them will be informative in a given laboratory cross.

Zebrafish Genetic Diversity

We used the program Structure v2.2.3 to evaluate the genetic diversity and ancestry of individuals of each strain (FALUSH *et al.* 2003; PRITCHARD *et al.* 2000). Structure can identify distinct genetic populations, assign individuals to populations, and assess individual shared ancestry. Figure S2 presents an analysis of the 2,875 SNPs positioned in the genetic map, within fish of the AB, IN, TL, WIK, C32, and SJD strains. We also included the grandparental G0 fish of the MGH cross (one AB, one IN) to assess how similar earlier samples of the AB and IN strains might be to those more recently obtained. We omitted TU from the analysis because sequence employed for SNP discovery was derived nearly exclusively from the TU strain (BRADLEY *et al.* 2007). Structure infers the allele frequencies of *K* ancestral populations on the basis of genotypes from a set of individuals and a user-specified value of *K*, and assigns a proportion of ancestry from each of the inferred *K* populations to each individual. The analysis was run without use of prior population information (omitting known strain identity), under a linkage model to accommodate linked

loci, varying *K* from 3 to 8. Ten replicate runs were made for each *K* to identify *K*=5 as a stable model at which the likelihood distribution reached a maximum. This was confirmed by 15 additional runs at *K*=5. The length of the burn-in period was 10,000, and the number of MCMC replications after burn-in was 10,000. The admixture burn-in length was 5,000 under the linkage model.

The resulting model is consistent with known ancestry. Present day laboratory strains of zebrafish are outbred, and are comprised of predominantly distinct genetic ancestry, shared across the strains to varying extents. The origins of the partially inbred lines C32 and SJD are visible in the data. C32 was derived from AB (Streisinger *et al.* 1981), and SJD was derived from the *Darjeeling* line (Nechiporuk *et al.* 1999), most related to the original MGH IN strain. Based upon net nucleotide distance of the predominant ancestral cluster of each strain, the most divergent pair of strains is AB and WIK. These observations contrast with lore among zebrafish researchers that AB and IN are most divergent (the reason that these were selected for the original MGH cross). However, the IN sample evaluated appears quite distinct from the original G0 IN grandparent of the cross. Values of Fst observed for the predominant ancestral clusters of AB, TL, IN, and WIK are each relatively high (0.86, 0.79, 0.79, and 0.77, respectively), to suggest relatively greater inter- than intra-strain variability in these managed laboratory populations. Fish of the partially inbred C32 and SJD lines that were evaluated had regions of remaining heterozygosity. For each, 11% of mapped clones contained heterozygous SNPs.

Literature Cited

Bradley, K. M., J. B. Elmore, J. P. Breyer, B. L. Yaspan, J. R. Jessen *et al.*, 2007 A major zebrafish polymorphism resource for genetic mapping. Genome Biol **8:** R55.

Falush, D., M. Stephens and J. K. Pritchard, 2003 Inference of population structure using multilocus genotype data: linked loci and correlated allele frequencies. Genetics **164:** 1567-1587.

Nechiporuk, A., J. E. Finney, M. T. Keating and S. L. Johnson, 1999 Assessment of polymorphism in zebrafish mapping strains. Genome Res **9:** 1231-1238.

Pritchard, J. K., M. Stephens and P. Donnelly, 2000 Inference of population structure using multilocus genotype data. Genetics **155:** 945-959.

Streisinger, G., C. Walker, N. Dower, D. Knauber and F. Singer, 1981 Production of clones of homozygous diploid zebra fish (Brachydanio rerio). Nature **291:** 293-296.
