# Supplementary material for: An SNP-Based Linkage Map for Zebrafish Reveals Sex Determination Loci
Source: G3 (Bethesda). 2011 Jun 1;1(1):3–9. doi: 10.1534/g3.111.000190 (PMC3178105; doi:10.1534/g3.111.000190)
Supplement: Supporting Information [file supp_1.1.3_000190SI.pdf]

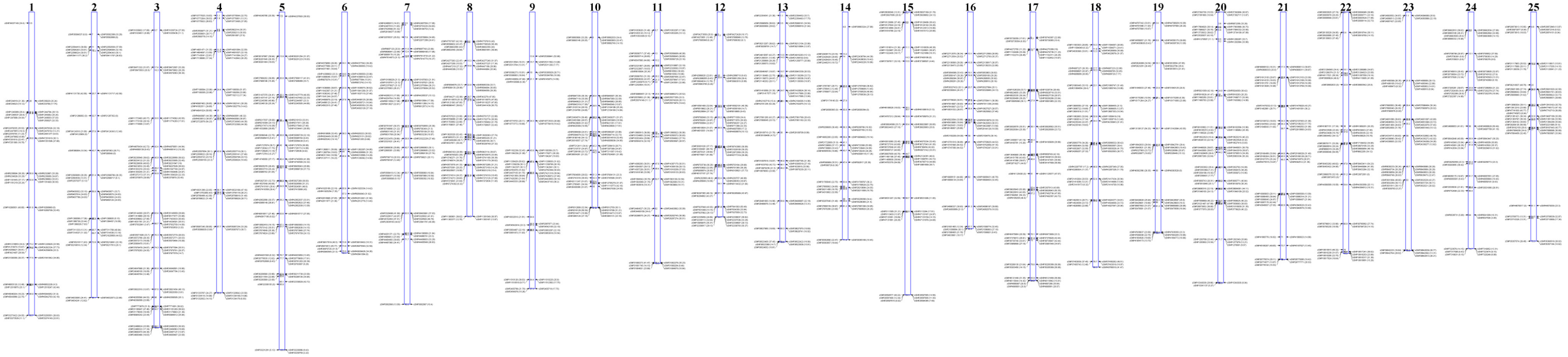

Figure S1 Zebrafish SNP mapping panel. SNPs that were optimally informative in all potential crosses of sampled fish of strain pairs, and that were optimally distributed across the genetic map were selected to form a generic SNP mapping panel for the zebrafish. The panel is comprised of 1,536 SNPs, capturing 519 unique loci with a mean locus density of 4.0 cM. The proportion of simulated crosses in which a given SNP was informative is noted adjacent to its identifier. Further detail of panel SNPs is given in Table S3.

High resolution image available at http://www.g3journal.org/lookup/suppl/doi:10.1534/g3.111.000190/-/DC1/FigureS1.pdf.

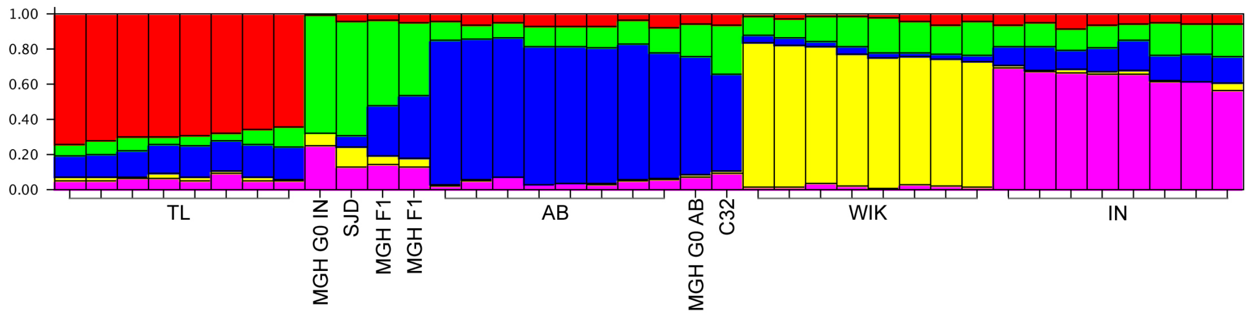

Figure S2 Estimated population structure of common laboratory zebrafish strains. Individual fish were genotyped for 2,875 genetically positioned SNPs for analysis by STRUCTURE under a linkage model. Each individual fish is represented by a vertical bar, which is partitioned into *K* colored segments that represent the estimated membership fraction from *K* ancestral populations. The highest probability model is presented, *K* = 5. Strain membership is designated at the bottom. The G0 grandparents (one AB, one IN) and F1 parents of the MGH cross are included for comparison. Prior information of strain membership was not employed in the analysis.

```
                                            L          K R    K                   P
Danio_rerio             1 1    ----MCFSVVSVVPLLFILWMLVVRFWRQSHRRTDGVAKENNKPEAVFPKLLHSLYKLFFSTVSPTISGPRSLPLLGNML 76
Tetraodon_nigroviridis  2 1    MSVEVLVAGVGAGFLLVLLLMVLKDMCVKKNHNL-----SFSAPPAVKRSLLQSLCGLLHRPSSPSIPGPPCRFLIGNMT 75
Fugu_rubripes           3 1    MVGKHLID--------------LDRMCSFLFCS------SFSAPPAVKRNLPLSLWQLPLRPSSPPIPGPPCRFLIGNMT 60
Gasterosteus_aculeatus  4 1    MVIEISFITAGAAFLVVLL-MIFIGIFCQKDQSV-----QGDHRGKAASGLLHHLCRFLTRSSSPGLPGPPSLFLIGNMM 74
Oryzias_latipes         5 1    ------------------------------------------------------------------------------MM 2
Anguilla_japonica       6 1    MPVCMTFVGAGTAVVLCVLWLVFVYYPRQTDQK---LCSQSN---VVGSTLLYPLNRLLPHTLSPSLPGPPSLPLLGNML 74
Homo_sapiens            7 1    MLL------LGLLLLLPLL---------------------------AGARLLWNWWKL------RSLHLPPLAPGFLHLL 41
Mus_musculus            8 1    MLL------PGLLLLLLLL---------------------------AGTRWLWGQWKL------RKLHLPPLAPGFLHFL 41

                                                                      H        R
                        1 77   DLAQDHLPIHLTALAKCYGNIYRLNCGSTTMVVLNNSEIIREALVKKWSDFAGRPYSYTGDIVSGGGRTISLGDFSEEWK 156
                        2 76   ELMQDHLPIHLTDLAKRYGNIYRLKCGNTTMVVLSSGDVIREALVKKWSDFAGRSVSYTADIVSGGGRTISLGDYTEEWK 155
                        3 61   ELMHDHLPIHLTNLAKRYGNIYRLKCGNTTMIVLNSSDIIREALVKKWSDFAGRAVSYTADIVSGGGRNISLGDYTEEWK 140
                        4 75   ELTHEHLPIHLTNLARRYGNIYRLKCGNTTMVVLNSGEIIREALVKKWSDFAGRPVSYTGDIVSGGGHTISLGDYNEEWR 154
                        5 3    ELMHDHLPIHLTSLAQRYGSIYRLKCGNTTMVVLNSGELIRETLVRKWSDFAGRPISYTGDIVSGGGRNISLGDYTEEWR 82
                        6 75   ELTRDHLPNHLTSLARHYGNIYRLYCGNTTIVVLSSAEMIREALVKKWSDFAGRPHSYTGDIVSGGGRSISLGDYSEAWR 154
                        7 42   Q---PDLPIYLLGLTQKFGPIYRLHLGLQDVVVLNSKRTIEEAMVKKWADFAGRPEPLTYKLVSRNYPDLSLGDYSLLWK 118
                        8 42   Q---PNLPIYLLGLTQKLGPIYRIRLGMQDVVVLNSNRTIEEALIQKWVDFAGRPHMLNGKMDL----DLSLGDYSLMWK 114

                        1 157  AHRRVTHSALQRCTTDSLHSVIEKQAQHLCQVLRDYSGKAVDLSEDFTVASSNVITTLTFSKAYDKSSAELQKLQECLNE 236
                        2 156  AHRRLVHSALQRCCKQSLHDVIERQALQLRKVLINYQGGAVDLSEDFTVAASNVITTLVFGKEYDKSCSELQQLHSCLNE 235
                        3 141  ALRRLVHGALQRCCKHSLHNVIERQALQLRKVLVDYRGGAVDLSEDFTVAASNVIITLVFGKEYDKSSSELQQLHRCLNE 220
                        4 155  AHRRLVHSALQRCCQQSLHDVIERQALHLREVLMDYRGSPVDLAEDFTVAASNVITTLAFGKEYGKSSSELQKLHSCLNE 234
                        5 83   AHRRLVHGALQRCCQQSLHSVIERQALHLRKVLMGYQGSPVDLSEDFTVAASNVITTLAFGKEYDKSSSELQQLHSCLNE 162
                        6 155  THRRLAHSALQRCSAQCLHAVIQKQALGLRQVLMDYNETPVDLSEDFTVAASNVITTLVFGKEYDKSSPELQRLHGCLNE 234
                        7 119  AHKKLTRSALLLGIRDSMEPVVEQLTQEFCERMRAQPGTPVAIEEEFSLLTCSIICYLTFGDKI-KDDNLMPAYYKCIQE 197
                        8 115  AHKKLSRSALMLGMRDSMEPLIEQLTQEFCERMRAQAGTPVAIHKEFSFLTCSIISCLTFGDK---DSTLVQTLHDCVQD 191

                                                                      -
                        1 237  IVSLWGSPWISALDSFPLLRKFPNPPFSRLMKEVARRDELIGKHIEEFKVRQIVQIISTESCQKFSKGGTLTSSLLKCLE 316
                        2 236  IVALWGSAWISALDTFPLLRKFPNPVFSRLLREVTRRDEIIRKHLNQYKVQS----------QDNKSTDVITGSLLEGVS 305
                        3 221  IVALWGSTWISALDTFPLLRKFPNPVFSRLLREVSRRDEIIRKHLNQFKVKS----------EGHRRTDVITGSLLEGVD 290
                        4 235  IVALWGSSWISALDSFPLLRKLPNPVFARLLKEVAKRDEIIREHLNNYK--S----------QNNKTEDAITGSLLEGLE 302
                        5 163  IVSLWGSSWISALDSFPLLRKLPNPVFARLLKEVARRDEIIKQHLDNFK--L----------QGHQSEDSITGSLLQALD 230
                        6 235  IVSLWGSPWISALDSFPLLRKLPNPPFSRLLKEVARRDDIIRSHIQEYK----------ELCQDEGTGGTITASLLQSLD 304
                        7 198  VLKTWSHWSIQIVDVIPFLRFFPNPGLRRLKQAIEKRDHIVEMQLRQHKESLV----------AGQWRDMMDYMLQGVAQ 267
                        8 192  LLQAWNHWSIQILTIIPLLRFLPNPGLQKLKQIQESRDHIVKQQLKRHKDSLV----------AGQWKDMIDYMLQGVEK 261

                                                                         M        H
                        1 317  PQQGAANHTTLTDTHVHMTTVDLLIGGTETIAALLNWTVAFLLHRPEVQDKVYEELCCVLDVR----YPQYSDRHKLPYL 392
                        2 306  KHR-KDHDVVLTDVHVHMATVDLLIGGTETTAAWLNWTVAFLLHRPEIQTKVYEELCTVLEGR----YPKYSDRHRLPVL 380
                        3 291  RHQ-TEPGVVLTDMHVHMATVDLLIGGSETTAAWLNWTVAFLLHRPEFQTKVYEELCTVLEGR----YPKYSDRQRLPIL 365
                        4 303  RNNNTDHKVRLTDIHVHMATVDLLIGGTETTAAWLNWTVAFLLHRPEVQAKVYEELCTVLEGR----YPKYSDRRRLPVL 378
                        5 231  KHQNTKEGQLLTDVHVHMATVDLLIGGTETTAAWLNWTVAFLLHRPEIQDKVYEELCTVPEGR----YPKYSDRQRLPVL 306
                        6 305  QPLGEKERVALTETHVHMATVDLLIGGTETTAAWLGWTVAFLLHRPEVQCRVYSELCTVLDTR----YPQYSDRLKLPCL 380
                        7 268  PXM-EEGSGQLLEGHVHMAAVDLLIGGTETTANTLSWAVVFLLHHPEIQQRLQEELDHELGPGASSSRVPYKDRARLPLL 346
                        8 262  QRD-GKDEERLHEGHVHMSVVDLFIGGTETTATTLSWAVAFLLHHPEIQKRLQEELDLKLGPG---SQLLYRNRMQLPLL 337

                        1 393  CALISEMLRLRPVAPLAVPHRAIRNSSIAGHFIPKNTIIIPNLYGAHHDPEVWDDPYSFKP--ERFLEGGGGSLRSLIPF 470
                        2 381  CSLVHEVLRLRPVAPLAVPHKAVRDSSIAGYFIPKNTIIIPNLFGAHHDPAVWPDPYSFKPVQERFLEGGGGSPRALVPF 460
                        3 366  CSLIHEVLRLRPVAPLAVPHKAIRDSSIAGYFIPRNTIIIPNLFGAHHDPEVWSDPYSFKP--ERFLEGGGGSTRALIPF 443
                        4 379  CSLINEVLRLRPVAPLAVPHRAVRDSSIAGYFIPKNTVIIPNLFGAHHDPAVWSDPYSFRP--ERFLEGGESSTRALMPF 456
                        5 307  CALVNEVLRLRPVAPLAVPHRAIRNSSIAGFFIPKNSVIIPNLFGAHHDPAVWSEPYSFKP--ERFLEGGGGSTRALVPF 384
                        6 381  SALINEVLRLRPVAPLAVPHRAIRDSSIAGYRIPKDTVIIPNLYGAHHDPAMWIDPYAFKP--ERFLDGGRASTRALLPF 458
                        7 347  NATIAEVLRLRPVVPLALPHRTTRPSSISGYDIPEGTVIIPNLQGAHLDETVWERPHEFWP--DRFLEP--GKNSRALAF 422
                        8 338  MATIAEVLRLRPVVPLALPHRATRASSISGYDIPKDMVIIPNIQGANLDEMVWELPSKFWP--DRFLEP--GKNPRTPSF 413

                                                                         A
                        1 471  GGGARLCLGEAVAKMEMFLFTAYLLREFKFLPASKEEPLPELRGV--ASVVLKVKPYTVIAHPR--EQ------- 534
                        2 461  GGGARLCLGETVAKMELFLFTAYLLRDFCFCLPDVEAALPDLRGV--ASVVLKVKAFTVVARPRIRPLS------ 527
                        3 444  GGGARLCLGETVAKMELFLFTAYLLRDFCFVLPDSEAPLPDLRGV--ASVVLKIKSFTVIARPR----------- 505
                        4 457  GGGARLCLGESVAKMELFLFTAYLLRDFHFIPPENEASLPDLRGV--ASVVLKVKSYTVTARPR--PLT------ 521
                        5 385  GGGARLCLGESVAKMELFLFTAYLLRDFQFILPESNVFPPDLRGI--ATVVLKIKPYKVIARPR--PAIGP---- 451
                        6 459  GGGARLCLGESIAKMELFLFTAYLLRDFQFLPSGNKETLPDLTGV--ASVVLKVKPFQVIVRPR--PLT------ 523
                        7 423  GCGARVCLGEPLARLELFVVLTRLLQAFT-LLPSGD-ALPSLQPLPHCSVILKMQPFQVRLQPRGMGAHSPGQSQ 495
                        8 414  GCGARVCLGEPLARLELFVVLARLLQAFT-LLPPPDGTLPSLQPQPYAGINLPIPPFQVRLQPRNLAPQDQGERP 487
```

Figure S3 Constraint-based multiple-alignment of 21-hydroxylase (cyp21a2). The cyp21a2 orthologs of *Danio rerio* (zebrafish), *Tetraodon nigroviridis* (green spotted pufferfish), *Fugu rubripes* (Japanese pufferfish), *Gasterosteus aculeatus* (stickleback),

Oryzias latipes (medaka), *Anguilla japonica* (Japanese eel), *Homo sapiens*, and *Mus musculus* are aligned for comparison. The male allele of *Danio rerio* identified within the cross is aligned at the top, with concurrent amino acid changes of the female allele designated above. Highlighted positions are allele changes at residues conserved among fish (in blue), and additionally among humans and mice (in yellow).

File S1

Supporting Text

Zebrafish SNP Mapping Panel

Each of the 2,854 positioned SNPs was evaluated to determine the proportion of potential crosses of pairs of fish in which the SNP would have been informative, among the genotyped fish of the AB, IN, TL, TU, WIK, C32, and SJD strains. These SNPs are provided in Table S2. Each simulated cross employed a pair of fish of different strains. To identify an optimal mapping panel of 1,536 SNPs, each positioned SNP was ranked according to the proportion of crosses in which it was informative. A first selection sweep was then made, in decreasing rank order, selecting each SNP that was no closer than 3 cM to a previously selected SNP. Subsequent iterative selection sweeps were then made (again in decreasing rank order), each sweep allowing single additional SNPs to be positioned within 3 cM of those SNPs added in prior sweeps (but at least 60 bp apart, to accommodate assay). Beyond the third iterative selection, to preclude addition of uninformative SNPs to a region already populated with ≥ 3 SNPs, only SNPs informative in at least 5% of the crosses were considered. However, for region populated with ≤ 2 SNPs, even a SNP uninformative in the simulated crosses but informative in the MGH cross (a validated SNP) was considered.

The resulting generic SNP mapping panel has a mean locus density of 4.0 cM (largest gap 26.2 cM), presented in Figure S1 and Table S3. These SNPs are well distributed and most informative for crosses of pairs of the common laboratory strains, based upon the fish sampled in our study. Within the mapping panel, each locus is typically redundantly represented by several SNPs to improve the probability that at least one of them will be informative in a given laboratory cross.

Zebrafish Genetic Diversity

We used the program Structure v2.2.3 to evaluate the genetic diversity and ancestry of individuals of each strain (Falush *et al.* 2003; Pritchard *et al.* 2000). Structure can identify distinct genetic populations, assign individuals to populations, and assess individual shared ancestry. Figure S2 presents an analysis of the 2,875 SNPs positioned in the genetic map, within fish of the AB, IN, TL, WIK, C32, and SJD strains. We also included the grandparental G0 fish of the MGH cross (one AB, one IN) to assess how similar earlier samples of the AB and IN strains might be to those more recently obtained. We omitted TU from the analysis because sequence employed for SNP discovery was derived nearly exclusively from the TU strain (Bradley *et al.* 2007). Structure infers the allele frequencies of *K* ancestral populations on the basis of genotypes from a set of individuals and a user-specified value of *K*, and assigns a proportion of ancestry from each of the inferred *K* populations to each individual. The analysis was run without use of prior population information (omitting known strain identity), under a linkage model to accommodate linked

loci, varying *K* from 3 to 8. Ten replicate runs were made for each *K* to identify *K*=5 as a stable model at which the likelihood distribution reached a maximum. This was confirmed by 15 additional runs at *K*=5. The length of the burn-in period was 10,000, and the number of MCMC replications after burn-in was 10,000. The admixture burn-in length was 5,000 under the linkage model.

The resulting model is consistent with known ancestry. Present day laboratory strains of zebrafish are outbred, and are comprised of predominantly distinct genetic ancestry, shared across the strains to varying extents. The origins of the partially inbred lines C32 and SJD are visible in the data. C32 was derived from AB (Streisinger *et al.* 1981), and SJD was derived from the *Darjeeling* line (Nechiporuk *et al.* 1999), most related to the original MGH IN strain. Based upon net nucleotide distance of the predominant ancestral cluster of each strain, the most divergent pair of strains is AB and WIK. These observations contrast with lore among zebrafish researchers that AB and IN are most divergent (the reason that these were selected for the original MGH cross). However, the IN sample evaluated appears quite distinct from the original G0 IN grandparent of the cross. Values of Fst observed for the predominant ancestral clusters of AB, TL, IN, and WIK are each relatively high (0.86, 0.79, 0.79, and 0.77, respectively), to suggest relatively greater inter- than intra-strain variability in these managed laboratory populations. Fish of the partially inbred C32 and SJD lines that were evaluated had regions of remaining heterozygosity. For each, 11% of mapped clones contained heterozygous SNPs.

Literature Cited

Bradley, K. M., J. B. Elmore, J. P. Breyer, B. L. Yaspan, J. R. Jessen *et al.*, 2007 A major zebrafish polymorphism resource for genetic mapping. Genome Biol **8:** R55.

Falush, D., M. Stephens and J. K. Pritchard, 2003 Inference of population structure using multilocus genotype data: linked loci and correlated allele frequencies. Genetics **164:** 1567-1587.

Nechiporuk, A., J. E. Finney, M. T. Keating and S. L. Johnson, 1999 Assessment of polymorphism in zebrafish mapping strains. Genome Res **9:** 1231-1238.

Pritchard, J. K., M. Stephens and P. Donnelly, 2000 Inference of population structure using multilocus genotype data. Genetics **155:** 945-959.

Streisinger, G., C. Walker, N. Dower, D. Knauber and F. Singer, 1981 Production of clones of homozygous diploid zebra fish (Brachydanio rerio). Nature **291:** 293-296.

Tables S1-S4 are available for download at http://www.g3journal.org/lookup/suppl/doi:10.1534/g3.111.000190/-/DC1.

Table S1 SNP and STR positions in the genetic map, and in the Zv9 physical assembly.

Table S2 SNPs of clones positioned in the genetic map, and observed alleles by strain.

Table S3 A mean 4 cM density SNP mapping panel for zebrafish. The panel was optimally informative within zebrafish of common laboratory strains sampled in the study (described in Supporting Text). SNP identifiers, genetic map position, and physical clone position are listed. An additional column provides the proportion of all possible crosses of the sampled fish in which each SNP would have been informative.

Table S4 Count and proportion of male and female F2 progeny in each genotypic category of the chromosome 5 and 16 sex-determination loci (see Figure 4).
